# Stable and Multilevel Data Storage Resistive Switching of Organic Bulk Heterojunction

**DOI:** 10.3390/nano11020359

**Published:** 2021-02-01

**Authors:** Harshada Patil, Honggyun Kim, Shania Rehman, Kalyani D. Kadam, Jamal Aziz, Muhammad Farooq Khan, Deok-kee Kim

**Affiliations:** 1 Department of Electrical Engineering, Sejong University, 209 Neungdong-ro, Gwangjin-gu, Seoul 05006, Korea; harshadapatil.nanotech@gmail.com (H.P.); khgking11@naver.com (H.K.); shania.rehman19@gmail.com (S.R.); kalyanikadam.nanotech@gmail.com (K.D.K.); azizjamal37@gmail.com (J.A.); 2 Department of Convergence Engineering for Intelligent Drone, Sejong University, Seoul 05006, Korea

**Keywords:** P3HT-PCBM, solution-processed, multilevel resistive switching, filament formation

## Abstract

Organic nonvolatile memory devices have a vital role for the next generation of electrical memory units, due to their large scalability and low-cost fabrication techniques. Here, we show bipolar resistive switching based on an Ag/ZnO/P3HT-PCBM/ITO device in which P3HT-PCBM acts as an organic heterojunction with inorganic ZnO protective layer. The prepared memory device has consistent DC endurance (500 cycles), retention properties (10^4^ s), high ON/OFF ratio (10^5^), and environmental stability. The observation of bipolar resistive switching is attributed to creation and rupture of the Ag filament. In addition, our conductive bridge random access memory (CBRAM) device has adequate regulation of the current compliance leads to multilevel resistive switching of a high data density storage.

## 1. Introduction

Recently, organic electronic devices have been considered to be a possible contender in the field of photovoltaics, sensors, and next-generation memory devices, due to their excellent performance and characteristics of organic materials [1,2,3,4]. Among them, organic nonvolatile memories have recently received significant attention because of their ease of fabrication, tunability of chemical structure, low power consumption, structural flexibility, solution processability, and high data storage [5,6,7,8]. Organic resistive switching memory has a simple structure composed of organic material as an active layer that sandwiches between two electrode materials. Notably, this active material is composed of polymers, organic small molecules, and hybrid organic–inorganic nanocomposites [9,10,11]. Investigations on such devices have also improved the resistive switching performance in terms of R_ON_/R_OFF_ ratio, retention time, multilevel data storage, and switching speed of the devices [12,13]. More recently, organic polymers have also been reported for neuromorphic computing due to their three-dimensional (3D) integration capability, low power consumption, and unique second-order memristive properties [14,15,16,17]. However, due to environmental sensitivity, poor stability, and low device reproducibility of organic materials, it has been serving together with inorganic materials [18]. To address these issues, many researchers have attempted to develop organic memory devices. Recently, Varun et al. reported the flexible resistive switching of PVP-GO composite with ultrathin HfO_2_ layer to harness the combined effect of composite material and thin oxide layer for better controllability [19].

Among the various organic materials, bulk heterojunction of P3HT-PCBM as a donor-acceptor material has gained attention, due to the high conductivity and efficiency of creating charges from photons in a nonvolatile memory device [20]. Although P3HT is a donor semiconducting polymer material, having strong coordination of hetero-sulfur atoms with metal ions would be beneficial for forming metallic filament [21]. In order to eliminate the problems regarding environmental stability and R_ON_/R_OFF_ ratio, the structure of organic and inorganic material has been employed. A native oxide layer on electrode material could intensify the performance of RRAM with respect to environmental stability, reliability, and R_ON_/R_OFF_ ratio. Frank et al. showed that the thin oxide layer of Al_2_O_3_ improved the device’s switching mechanism and reliability [22]. Zinc oxide (ZnO) has proven its potential capability in RRAM performance due to its large band gap (3.37 eV) and high exciton binding energy (B.E) (60 MeV) at absolute temperature. ZnO has been widely considered for unipolar and bipolar resistive switching, i.e., Al/ZnO/Al, Ti/ZnO/Pt, and Ti/CeO_2_/ZnO/Pt to improve the performance of resistive switching behavior [23,24,25]. We incorporate ZnO nanoparticles as a capping protective layer of bulk heterojunction, which further provide better control over the filament formation and rupture and increase the ON/OFF ratio. In addition to the stable operating device requirement, the device also needs high-density data storage. Multilevel memory provides a unique opportunity to store more than two data levels in a single device [26]. However, in P3HT-PCBM bulk heterojunction, the multilevel data storage has not yet been illustrated. Our report’s main aim is to improve environmental stability, high ON/OFF ratio, and multilevel resistive switching by using Ag/ZnO/P3HT-PCBM/ITO structure.

## 2. Materials and Methods

Indium tin oxide (ITO) coated glass substrates were obtained from AMG, the Republic of Korea, and the dry powders of P3HT and PCBM were bought from Solaris Chem Technology, Canada. To make the bulk heterojunction of P3HT-PCBM, it was dissolved in dichlorobenzene with a mass ratio of 1:0.8 at a total concentration of 27 mg/mL. This prepared solution was kept for stirring at 65 °C, for 12 h. The ZnO stock solution with 20 wt% (Ditto Technology, Republic of Korea) was diluted in methanol solution at 3 wt% ZnO. The ITO-coated glass substrates were washed with detergent for the device fabrication and subsequently washed in an ultrasonic cleaner for 10 min with distilled water, acetone, and isopropyl alcohol. The prepared mixture of P3HT-PCBM was spun coated on the ITO substrate with PTFE (0.45 µm) filter at 1000 rpm for 120 s. The soft baking of film was kept in a conventional oven for 10 min at 110 °C for drying purpose. After the annealing of film, the ZnO solution was coated on prepared bulk-heterojunction film at 5000 rpm for 60 s, and kept in the oven for 5 min at 110 °C. The top electrode Ag with 100 nm thickness was deposited in a vacuum thermal evaporator via a metal shadow mask, with an annular pattern range between 80 and 200 µm diameter. All the electrical measurements were taken using an Agilent B1500 semiconductor parameter analyzer in an atmospheric environment. The top electrode (Ag) was positively biased while the bottom electrode (ITO) was grounded during measurements.

## 3. Results and Discussion

**Figure 1:**
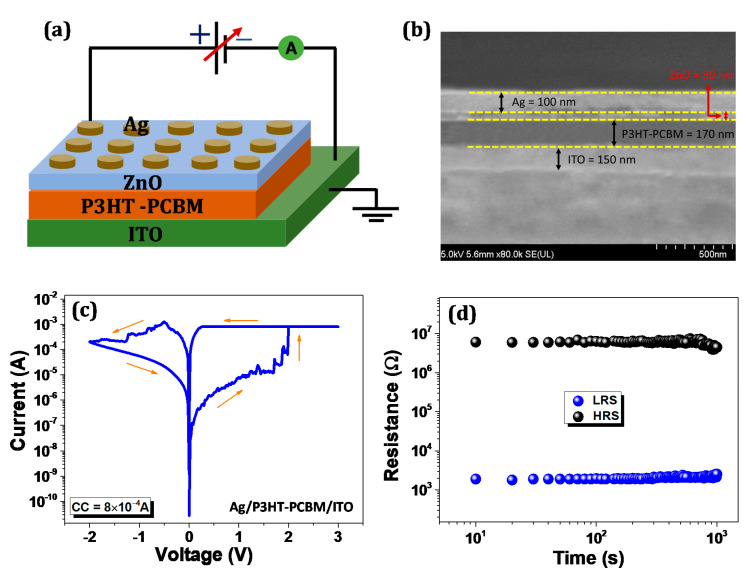
(**a**) Schematic layout of fabricated device with the measurement configuration; (**b**) Cross-sectional SEM image of sandwich structure; (**c**) Typical I-V curve of Ag/P3HT-PCBM/ITO in semi-logarithmic scale; (**d**) Retention characteristic in both high resistance state (HRS) and low resistance state (LRS).

Figure 1a describes the schematic layout of the Ag/ZnO/P3HT-PCBM/ITO fabricated device with measurement system. A cross-sectional SEM image of the sandwiched structure, in Figure 1b, shows that the film thickness of the organic and inorganic protective layers are about 170 and 30 nm, respectively. Figure 1c shows the typical I-V structure of Ag/P3HT-PCBM/ITO in semi-logarithmic scale, which belongs to bipolar resistive switching behavior. It is interesting to note that the conventional electroforming process was not applied to the device during I-V measurements. The electroforming free operation of the resistive memory device scale down the complexity of the memory circuit and the expense of the memory system. Moreover, the electroforming free process improves power consumption and device footprint [27]. In the first 0 to 3 V voltage sweep, the current is gradually increasing, due to the inherent conductivity of the organic layer and, at 1.9 V, the current shows an abrupt change, indicating the ”SET” process from high resistance state (HRS) or OFF state to low resistance state (LRS) or ON state. At positive bias, current compliance (CC) was added to shield the device from breakdown.

When the negative voltage sweeps biased, the device returns to its original state HRS, thus, indicating “RESET” process from LRS to HRS. However, in organic bulk heterojunction, the ON/OFF ratio is maintained at 10^2^–10^3^. Figure 1d shows the device’s retention characteristics; the device shows the degradation of resistance in LRS and HRS after 10^3^ s. This designates that the reliability of organic memory has not been effectively maintained. Thus, the adsorption of water and oxygen from the atmosphere in organic active layer causes the degradation of organic memory [28]. The main issue that arises in bulk heterojunction is the atmospheric stability of the device. Therefore, modifying the memory architecture is the key to modern organic resistive switching devices.

**Figure 2:**
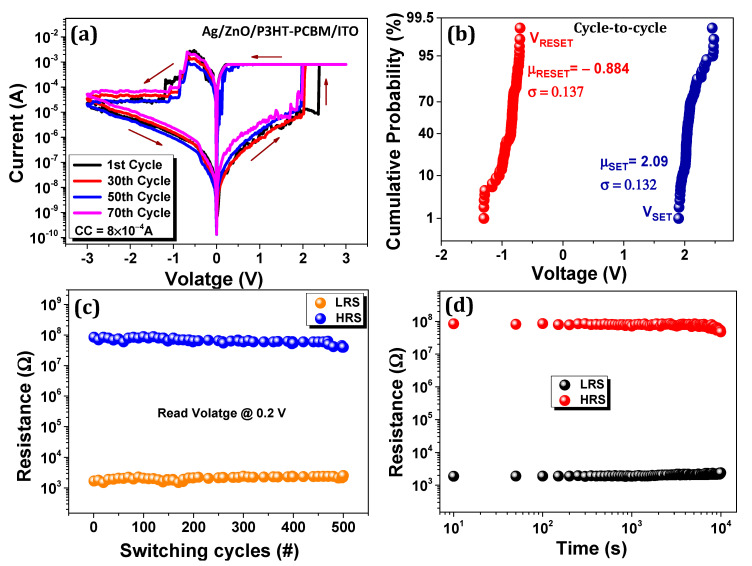
(**a**) Typical I-V characteristic of Ag/ZnO/P3HT-PCBM/ITO in semilogarithmic plot. (**b**) Cumulative probability of SET/RESET voltages; (**c**) DC sweep mode endurance cycles at read voltage 0.2 V, respectively; (**d**) Retention property in both LRS and HRS at read voltage 0.2 V.

We proposed a new device, i.e., Ag/ZnO/P3HT-PCBM/ITO based on the above discussion. All the electrical properties were examined under room temperature. Figure 2a illustrates that bipolar resistive switching is exhibited in the measured I-V curve under a compliance current of 800 µA. The voltage was applied to the top electrode Ag, and the bottom electrode ITO was grounded to measure the resistive switching phenomenon of the device. No forming voltage was necessary for our device; it is good to meet for practical application. ZnO acts as a protective layer which also prevents the interfacial reactions and decreases the charge carrier injection at electrode interface [29]. When voltage is applied to positive polarity, the device shows a gradual increase in current from 0.2 to 1.9 V, due to intrinsic mobility of materials in the device, and abrupt change varies from 1.9–2.3 V, which designates the device transit from HRS to LRS. When the reverse voltage is applied, the current decreases gradually and, at the RESET, voltages vary from V_RESET_ > 0.7 to 1.2 V due to the random rupture of filaments [19].

The asymmetry of the current levels in both polarities occurs due to the different work function of two electrodes and materials [30]. The statistical distribution is one of the crucial memory parameters in memory devices. Figure 2b shows the cumulative probability of V_SET_ and V_RESET_ voltages of 100 DC switching cycles for the ZnO layer device. The coefficient of variation (CV) is defined as the ratio of standard deviation (σ) to the mean (µ). The CV of V_SET_ and V_RESET_ voltages are 6.3% and 15.4%, respectively. In the SET process, superior uniformity was obtained while the RESET voltage variance is remarkable, which could be due to the creation and rupture process of Ag atoms’ conductive filament [31]. Furthermore, in Supplementary Materials Figure S1, we estimate the cumulative probability based on the resistance level of 20 different devices with their different structures, showing a wider memory window (10^5^) with ZnO layer than that without ZnO layer (10^3^). The CV of LRS and HRS with ZnO layer are 10.5% and 17.9%, while the CV without ZnO layer are 66.2% and 79.5%, respectively. Therefore, clearly, the device with ZnO layer can give better control on conductive filament. Figure 2c depicts DC endurance cycles in which the LRS and HRS show stable performance up to 500 switching cycles and a resistance ratio of about 10^5^. Figure 2d represents the retention, which confirms the device’s stability at 0.2 V read voltage. The resistance in LRS is slightly shifted towards the upper side, but the overall ON/OFF state is well maintained up to 10^4^ s. During this measurement, the device exhibits a high ON/OFF ratio 10^5^.

**Figure 3:**
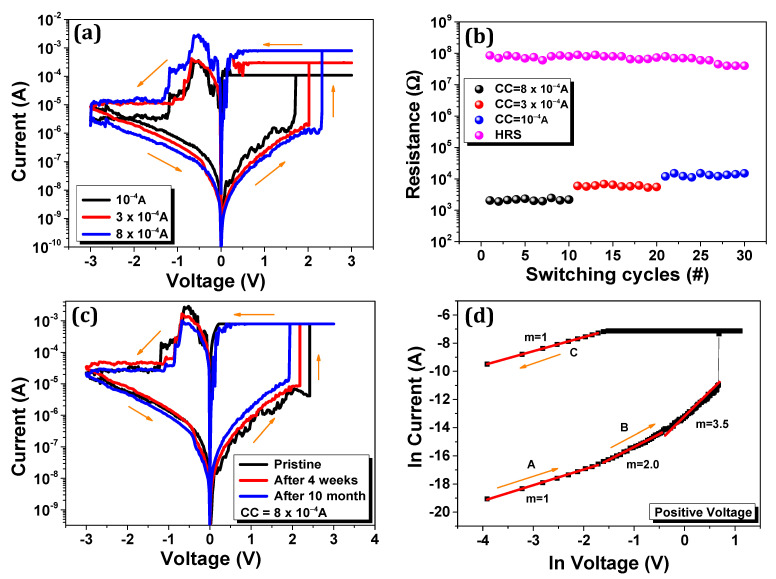
(**a**) I-V characteristics of memory device under different compliance current of 100, 300, and 800 µA, consecutively; (**b**) Multilevel resistance states with distinct current compliance of 800, 300, and 100 µA, consecutively; (**c**) Electrical characterization of the Ag/ZnO/P3HT-PCBM/ITO device after storage in ambient air; (**d**) Double logarithmic I-V curve of Ag/ZnO/P3HT-PCBM/ITO bulk-heterojunction device, illustrating the slope values of linear fitted curve at positive bias. The plot divided into three different regions represented as segment A, segment B, and segment C, respectively.

Furthermore, we also demonstrate the multilevel data storage that stores the data and enhances the memory capacity, as shown in Figure 3a. We applied the compliance current at 100, 300, and 800 µA, consecutively. During the multilevel data storage, HRS was unaffected, but the LRS expanded as the current compliance declined. This outcome shows that the LRS can be tuned to the SET phase by a different compliance current. Figure 3b depicts the number of cycles to illustrate the multilevel resistive switching; the states of resistance were determined during 0.2 V read voltage and CC at 800, 300, and 100 µA, consecutively. The four data levels with the one stable HRS and three levels of LRS were achieved in multilevel switching. Each multilevel resistive state revealed different resistance levels for ten cycles.

Materials need to be durable to illustrate the practical applications of electronic devices. Most organic bulk heterojunctions are stable under ambient conditions and prepared, stored under an N_2_ atmosphere [32,33,34]. According to a previous study, to achieve high stability and reliability of the device, a thin metal oxide layer may be used as a protective layer [35]. To assess the prolonged stability, we stored the device in a surrounding atmosphere at 28–30 °C, with 70% humidity. We measured the device in the pristine state, and after several weeks, the resistive switching properties did not show any prominent degradation of the device, as shown in Figure 3c. These results specify that in practical applications, bulk heterojunction with inorganic protective layer can be used.

**Figure 4:**
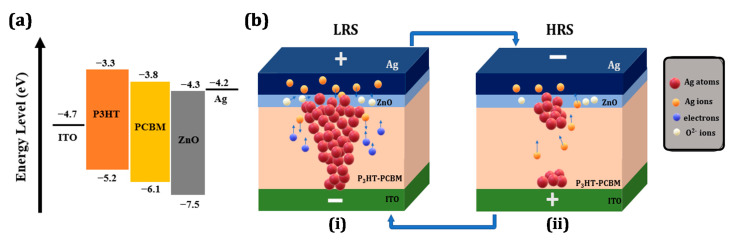
(**a**) Energy band diagram of proposed device structure. (**b**) Schematic presentation of resistive switching mechanism in HRS and LRS, respectively.

The conduction mechanism of bulk heterojunction and oxide layer resistive memory device Ag/ZnO/P3HT-PCBM/ITO was analyzed by ln of I-V curve and with the aid of the minimal space charge limited current (SCLC) mechanism [36], as shown in Figure 3d. Segment C demonstrates the LRS state, i.e., the SET process and follows the current’s linear action against voltage I α V; m ~ 1, showing the metallic existence of the conduction filament. Whereas in HRS, segment A presents a low positive region that confirms the Ohmic conduction I α V with slope value 1; due to the thermally produced free charge carriers and low current flow amounts, this conduction occurs. When the applied voltage increases, the square law dependence of current I α V^2^, with the slope value of m = 2 is demonstrated. This shows that the device in its HRS demonstrates SCLC conduction mechanism. In contrast, the LRS is associated with the formation of metallic filament. The ZnO and P3HT-PCBM can be shown in the form of the highest occupied molecular orbit (HOMO) and the lowest unoccupied molecular orbit (LUMO), as presented in Figure 4a [37]. When DC voltages are applied to the top electrode, electrons can move easily from the LUMO level of the ZnO to the LUMO level of the P3HT-PCBM, and therefore, due to the different energy levels in materials and electrodes, it has asymmetry behavior in current levels of both polarities.

On the basis of the above discussion, we propose a probable resistive switching phenomenon in resultant devices with a scientifically acceptable circumstances of formation and creation of conductive filament in localized region. As shown in Figure 4b, (i) an active Ag electrochemical electrode is oxidized into Ag^+^ ions when a positive voltage is applied to the top electrode, and under the high electric field, these ions start migrating towards the bottom electrode. Thus, electrons generated from the bottom electrode move up towards the top electrode. In this procedure, down-transferring Ag^+^ ions are reduced by up moving injected electrons to Ag atoms. Eventually, Ag atoms start to form a conductive filament path from the bottom to the top electrode, due to the reduction of Ag^+^ ions. The conductive filament formation results in the saltation of current and the device transforms from HRS to LRS, called “SET” process. However, the presence of ZnO will also initiate some oxygen vacancies, which can also aid in conductive filament formation. However, the possibility of Ag filament formation is higher [33,38,39]. In order to back, a negative voltage is applied device back to HRS is known as the “ERASE” process. A conductive filament breakdown aided by joule heating was observed; this is due to the excess amount of current flow through the tiny conductive filament. Moreover, the Ag^+^ ions pile up to the top electrode and again they reduced into Ag, as shown in Figure 4b (ii).

## 4. Conclusions

In summary, we fabricated low cost and solution-processed organic bulk heterojunction with inorganic capping material for a resistive switching device. The cross-sectional SEM confirms the prepared layered structure of the device. The fabricated device exhibited reliable and reproducible bipolar resistive switching with excellent I_ON_/I_OFF_ ratio of 10^5^, retention of 10^4^ s, and DC endurance of 500 cycles. The multilevel resistive switching to level four data storage efficiency is demonstrated by the application of various compliance current to memory devices, demonstrating the high data density memory application. A thin metal oxide layer acts as a capping layer, which further promotes better memory application efficiency. The conduction mechanism of the fabricated device is based on the filament creation and rupture of Ag metal. According to the above findings, the proposed device has potential as a candidate for high performance RRAM with air-stable and multilevel data storage.

## Data Availability

Data is contained within the article and supplementary material.

## Supplementary Materials

The following are available online at https://www.mdpi.com/2079-4991/11/2/359/s1, Figure S1: cumulative probability based on the HRS and LRS of 20 different devices with their different structures.
