# Supplementary material for: Stable and Multilevel Data Storage Resistive Switching of Organic Bulk Heterojunction
Source: Nanomaterials (Basel). 2021 Feb 1;11(2):359. doi: 10.3390/nano11020359 (PMC7912748; doi:10.3390/nano11020359)
Supplement: Supplementary file 1 [file nanomaterials-11-00359-s001.pdf]

Supplementary Information

Stable and multi-level data storage resistive switching of organic bulk-heterojunction

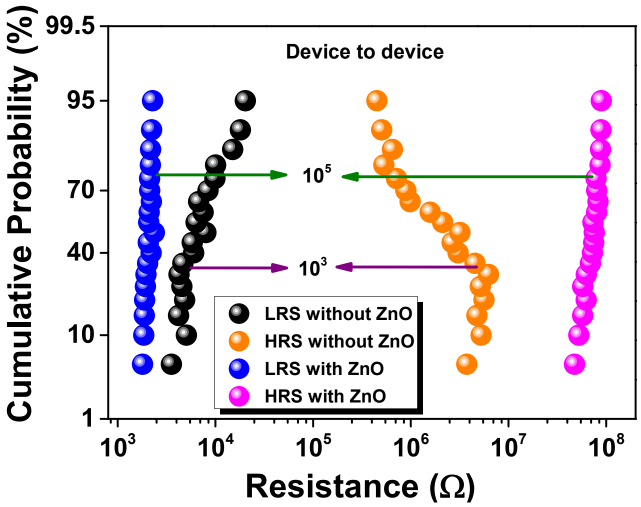

Figure S1: Cumulative probability based on the HRS and LRS of 20 different devices with their different structures.

The cumulative probability based on the resistance states of 20 different devices with their different structures, it has shown a wide memory window (10^5) with ZnO layer than the without ZnO layer (10^3). The Coefficient of variance (CV) of LRS and HRS with ZnO layer are 10.5% (σ/μ = 222.0/2113) and 17.9% (σ/μ = $1.32\times10^7/7.38\times10^7$), while CV of without ZnO layer are 66.2% (σ/μ =5922/8936) and 79.5 % (σ/μ = $2.28\times10^6/2.87\times10^6$), respectively.
